# Supplementary material for: Primary Ciliary Dyskinesia and Retinitis Pigmentosa: Novel RPGR Variant and Possible Modifier Gene
Source: Cells. 2024 Mar 16;13(6):524. doi: 10.3390/cells13060524 (PMC10968961; doi:10.3390/cells13060524)
Supplement: Supplementary file 1 [file cells-13-00524-s001.zip › Baz-Redon_Supplementary_Materials.pdf]

Primary ciliary dyskinesia and retinitis pigmentosa: novel *RPGR* variant and possible modifier gene

Noelia Baz-Redón, Laura Sánchez-Bellver, Mónica Fernández-Cancio, Sandra Rovira-Amigo, Thomas Burgoyne, Rai Ranjit, Virginia Aquino, Noemí Toro-Barrios, Rosario Carmona, Eva Polverino, Maria Cols, Antonio Moreno-Galdó, Núria Camats-Tarruella, Gemma Marfany

SUPPLEMENTARY MATERIALS AND METHODS

Primary ciliary dyskinesia diagnostic evaluation

Following the European Respiratory Society (ERS) guidelines for PCD diagnosis [1] and in-house optimised protocols, the ciliary function and structure in the proband, siblings and mother were evaluated with a clinical symptoms questionnaire and PICADAR (PrImary CiliARy DyskinesiA Rule) score [2], nasal nitric oxide (nNO) screening test, high-speed video-microscopy analysis (HSVM), immunofluorescence (IF) [3] and transmission electron microscopy (TEM) analyses and genetics [4]. Nasal brushing sample from the father was unavailable, as he was deceased.

Nasal nitric oxide (nNO) screening

nNO measurements were performed using a CLD 88sp NO-analyzer (ECO MEDICS, AG, Duerten, Switzerland). A recent ERS technical standardisation recommends a cut-off of 77nL/min for nNO measurements during breath-hold manoeuvres [5].

High-speed video-microscopy analysis (HSVM)

HSVM was performed to study ciliary beat frequency (CBF) and ciliary beat pattern (CBP). Local normal values: CBF 8.56-11.32 Hz; CBP ≤20% dyskinetic ciliated cells (*unpublished data*). Nasal respiratory epithelia at the inferior nasal meatus were sampled with a 2 mm diameter brush submerged in HEPES-supplemented Medium 199. A minimum of ten lateral strips with 10 cells each and two overhead axes were captured at 37°C with an optical microscope coupled to a high-speed video camera (MotionPro® X4, IDT, CA, USA) using MotionPro® X4 software [6].

Immunofluorescence (IF) analysis

IF was performed from nasal-brush respiratory epithelial samples, which were dropped, air dried, and stored at -80°C until use. Cells were prepared and incubated with primary antibodies anti-DNAH5 (an ODA component), anti-DNALI1 (an IDA component), anti-GAS8 (a nexin-DRC component) and anti-RSPH9 (a radial spoke head component), following previously reported methodology and analysis [3].

Transmission electron microscopy (TEM) analyses

Nasal brushing samples were fixed in 2.5% glutaraldehyde in cacodylate buffer for more than 24 hours before being centrifuged in 2% agar to generate pellets. Subsequently, samples were postfixed in 1% osmium tetroxide for 1 hour. These pellets were then en bloc stained in undiluted UA-Zero (Agar Scientific, Agar Scientific Ltd, Stansted, UK) for 30 minutes at room temperature. Using increasing

concentrations of ethanol followed by propylene oxide, the pellets were dehydrated before embedding in Araldite resin. 80 nm thick sections were cut and imaged using a JEOL 1400+ transmission electron microscope (TEM) fitted with an AMT 16X CCD camera.

Genetics

Peripheral blood DNA was extracted by automated magnetic extraction (Chemagic, Perkin-Elmer, Waltham, MA, USA). The DNA concentration was determined with a Qubit dsDNA BR Assay Kit in a Qubit 2.0 fluorimeter (Thermofisher, Waltham, MA, USA).

To carry out the genetic studies, a high-throughput gene panel was designed to allow exonic and flanking intronic region sequencing using KAPA HyperCap Target Enrichment technology from Roche Diagnostics (Roche Sequencing Solutions, Pleasanton, CA, USA). We used an amplified version of our custom gene panel [4], which include 104 genes related to PCD, as described in the literature at time of design. DNA libraries with the regions of interest were prepared according to the manufacturer's instructions and sequenced in a MiSeq platform (Illumina, San Diego, CA, USA).

Whole Exome Sequencing (WES) using Agilent SureSelect Human All Exon v6 (Agilent Technologies, Santa Clara, CA, USA) and sequenced in a NovaSeq600 Illumina platform was performed to deeply analyse genotypic variability among the family members.

Obtained sequence reads were aligned to the human reference sequence hg38 in the case of the gene panel and hg19 in the WES. Duplicated reads were removed with Picard tools. Local realignment, recalibration, and variant calling were conducted with the Genome Analysis Tool Kit (GATK version 4.1.2). Only variants with a deep-coverage, i.e. ≥20 reads, were taken into account in the analysis.

Variants with minor allele frequencies >1% in the 1000genomes browser (http://phase3browser.1000genomes.org/index.html), NHLBI Exome Sequencing Project Exome Variant Server (http://evs.gs.washington.edu/EVS/), and Genome Aggregation Database (http://gnomad.broadinstitute. org/) were excluded. The list of the identified variants was compared with information from different databases to identify variants already described in association to a known phenotype: HGMD (Qiagen, Hilden, Germany), ClinVar (https://www.ncbi.nlm.nih.gov/clinvar/) [7]. The pathogenicity of the variants was evaluated using Varsome (Saphetor, Lausanne, Switzerland) [8]. The sequencing data were reanalysed using the bioinformatics software ExomeDepth v1.1.15 [9] to detect copy number variations (CNVs). Additionally, structural variants were analysed in WES using Gridss (v2.13.2) software [10]. The nomenclature and classification of variants was based on the Human Genome Variation Society (HGVS) guidelines (https://www.hgvs.org/) [11] and the American College of Medical Genetics and Genomics (ACMG) (https://www.acmg.net/) [12]. Sequence variants were classified into five categories: pathogenic, likely pathogenic, variants of uncertain clinical significance (VUS), likely benign, and benign, according to ACMG [12]. Pathogenic, likely pathogenic and VUS variants were confirmed by Sanger sequencing using custom-designed primers.

In vitro expression and functional studies

Plasmid vectors and plasmid constructions

pEGFP-LAP-RPGR$_{Ex1-19}$ was a kind gift from Dr. Sheffield [13]. Also, an empty vector pEGFP-C2 (Clontech Laboratories, Takara Bio, Kusatsu, Japan) was used as transient transfection control.

To generate the pEGFP-LAP-RPGR$_{Ex1-19}^{T307K}$ (c.920C>A; p.T307K) mutant vectors, a two-step PCR strategy described in Cols *et al.* [14] was followed. Briefly, a first PCR was carried out using the AccuPrime™ Taq DNA Polymerase, High Fidelity (Thermo Fisher Scientific, Rockford, IL, USA; 12346086) with primers 5′-AGCTCCTGGGCAATCACAG-3′, which amplified from a Kpn2I restriction site, and 5′-CTGTTATCAAAGCTTTGTGATTTTCTCCAC-3′, which introduced the desired point mutation. The resulting PCR product was purified with the NZYGelpure Kit (NZYTech – Genes&Enzymes, Lisbon, Portugal; MB01101), according to the manufacturer's protocol. Then, the amplicon was used as forward primer together with the Psp5II restriction site-containing reverse primer 5′-AATCTGGTTCCTCTGGCTG-3′ to amplify a longer PCR product (flanked by Kpn2I and Psp5II target sequences) at 45°C for primer annealing. After DNA purification with the NZYGelpure Kit (NZYTech – Genes&Enzymes, Lisbon, Portugal; MB01101), both the product from the second PCR and the plasmids encoding the WT RPGR open reading frame (ORF) were digested with FastDigest Psp5II and FastDigest Kpn2I (Thermo Fisher Scientific, Rockford, IL, USA; FD0764 and FD0534) for 1 hour at 37°C and the DNA bands were collected by purification from agarose gel again with the NZYGelpure Kit (NZYTech – Genes&Enzymes, Lisbon, Portugal; MB01101). Ligation of the insert and the destination vector was performed using T4 DNA ligase (Thermo Fisher Scientific, Rockford, IL, USA; EL0011) overnight at 17°C, and the ligation product was transformed into DH5α *E.coli* bacteria (Thermo Fisher Scientific, Rockford, IL, USA; 18265017) by heat-shock. Plasmid purification was conducted with QIAprep Spin Miniprep Kit (Qiagen, Germantown, MD, USA; 27104) following manufacturer's instructions. Finally, Sanger sequencing was performed to verify correct mutagenesis, sequence integrity, orientation of the inserts and reading frame.

Cell culture and plasmid transient transfections

Human retinal pigment epithelial cells (hTERT-RPE1) cells were cultured in 10% foetal bovine serum (FBS) (Life Technologies, Carlsbad, CA, USA) and 1% penicillin/streptomycin (Life Technologies, Carlsbad, CA, USA) in 1:1 Dulbecco's Modified Eagle's Medium (DMEM) (ATCC, Manassas, VA, USA) and Ham's F-12 Nutrient Mix (F12) (Life Technologies, Carlsbad, CA, USA) in a 5% CO2 cell culture humidified incubator at 37°C. For ciliogenesis induction, hTERT-RPE1 cells were serum-starved with 0.2% FBS and 1% penicillin/streptomycin in 1:1 DMEM:F12 for 24 hours.

Empty vector (pEGFP-C2), WT plasmid (pEGFP-LAP-RPGR$_{Ex1-19}$) and mutant plasmid (pEGFP-LAP-RPGR$_{Ex1-19}^{T307K}$) were transiently transfected. Cells in suspension (2x10^5 cells/well on coverslips in 24-well plates) were transfected using 500 ng of plasmid DNA and Lipofectamine 2000 (Invitrogen, Carlsbad, CA, USA; 11668027) (DNA–Lipofectamine ratio 1:2) in non-antibiotic medium, and 4 hours post-transfection, the non-antibiotic medium was replaced with fresh complete medium which was then re-placed with starvation medium, 24 hours after transfection.

Immunofluorescence analyses of RPGR and CEP290 in nasal brushing samples

Respiratory epithelial cells from nasal brushing biopsies on glass slides were fixed in 4% paraformaldehyde (PFA) for 15 minutes followed by three washes with phosphate-buffered saline

(PBS). Permeabilisation was carried out with 0.2% Triton X-100 (Sigma-Aldrich, St. Louis, MO, USA) in PBS for 20 minutes at room temperature. Next, the slides were incubated with 5% bovine serum albumin (BSA) in PBS with 0.1% Triton X-100 (Sigma-Aldrich) for 1 hour at room temperature. The primary antibodies: anti-γ-TUBULIN (Sigma-Aldrich, T6557, 1:500) and anti-RPGR (Sigma-Aldrich, HPA001593, 1:100) or anti-CEP290 (Thermo Fisher Scientific, Rockford, IL, USA; A301-659A; 1:100) were incubated overnight at 4°C in blocking solution. After two washes with PBS, samples were incubated with the corresponding secondary antibodies in blocking solution for 1 hour at room temperature: AlexaFluor 488 anti-Rabbit (Thermo Fisher Scientific, A11070, 1:300), AlexaFluor 647 anti-Mouse (Thermo Fisher Scientific, A21235, 1:300). Nuclei were stained with 4′,6-diamidine-2′-phenylindole dihydrochloride (DAPI) (Sigma-Aldrich, 10236276001, 1:1000). A 2-hour incubation at room temperature was used for AlexaFluor 546-conjuaged acetylated α-TUBULIN (Santa Cruz Biotechnology, Dallas, TX, USA; sc-23950 AF546; 1:250). Finally, the slides were rinsed and coverslipped with ProLong™ Gold Antifade Mountant (ThermoFisher Scientific; P1014).

Immunofluorescence analyses in hTERT-RPE1 cells

Forty-eight hours post-transfection, hTERT-RPE1 cells were fixed in 2% PFA for 5 minutes followed by 4% PFA for 30 minutes at room temperature. Afterwards, cells were rinsed with PBS, permeabilised in 0.5% Triton X-100 (Sigma-Aldrich, St. Louis, MO, USA) in PBS for 20 minutes at room temperature, and blocked for 1 hour in 10% Normal Goat Serum (NGS) (Thermo Fisher Scientific, Rockford, IL, USA; 16210064) in PBS with 0.2% Triton X-100 (Sigma-Aldrich, St. Louis, MO, USA). Anti-γ-TUBULIN (Sigma-Aldrich, St. Louis, MO, USA; T6557; 1:500) and anti-GFP (Abcam, Plc, Cambridge, UK; ab290; 1:500) primary antibodies were incubated overnight at 4°C in blocking solution. After incubation, cells were washed with PBS and incubated with the secondary antibodies in blocking solution for 1 hour at room temperature: AlexaFluor 488 anti-Rabbit (Thermo Fisher Scientific, Rockford, IL, USA; A11070; 1:300), AlexaFluor 647 anti-Mouse (Thermo Fisher Scientific, Rockford, IL, USA; A21235; 1:300). Nuclei were stained with 4′,6-diamidine-2′-phenylindole dihydrochloride (DAPI) (Sigma-Aldrich, St. Louis, MO, USA; 10236276001; 1:1000). Next, a 2-hour incubation at room temperature was used for AlexaFluor 546-conjugated acetylated α-TUBULIN (Santa Cruz Biotechnology, Dallas, TX, USA; sc-23950 AF546; 1:250). Finally, coverslips were rinsed and mounted using Mowiol 4-88 (Merck, Kenilworth, NJ, USA).

Microscope image acquisition and analysis

Samples were analysed by confocal microscopy (Zeiss LSM 880, Thornwood, NY, USA) and images were collected using ZEN-LSM software (version 2.3, Zeiss, Thornwood, NY, USA). The ImageJ software (National Institutes of Health, Bethesda, MD, USA) was used for processing, measurement and analysis of confocal immunofluorescence images. Fluorescence intensity plot profiles were generated from one representative confocal immunofluorescence image per condition. RPGR and CEP290 fluorescence intensities were measured using the integrated density parameter and then the corrected total cell fluorescence formula [CTCF = Integrated density of desired region – (Area of desired region x Mean fluorescence of 3 background readings)]. The distribution of RPGR and CEP290 in the different subcompartments of multiciliated epithelial nasal cells (transition zone and cytoplasm) was estimated by dividing the CTCF value of the subcompartment of interest by the total CTCF value of the cell.

Cilia length was measured from the base of the cilium (immunolabelled with anti-γ-tubulin) to the tip of the axoneme (immunostained with anti-acetylated α-tubulin) in GFP-positive cells. For quantitative analysis of ciliogenesis, the percentage of ciliated cells versus non-ciliated cells was counted manually, considering only the GFP-positive cells.

References:

1. Lucas, J.S.; Barbato, A.; Collins, S.A.; Goutaki, M.; Behan, L.; Caudri, D.; Dell, S.; Eber, E.; Escudier, E.; Hirst, R.A.; et al. European Respiratory Society Guidelines for the Diagnosis of Primary Ciliary Dyskinesia. *Eur. Respir. J.* **2017**, *49*, 1601090, doi:10.1183/13993003.01090-2016.
2. Behan, L.; Dimitrov, B.D.; Kuehni, C.E.; Hogg, C.; Carroll, M.; Evans, H.J.; Goutaki, M.; Harris, A.; Packham, S.; Walker, W.T.; et al. PICADAR: A Diagnostic Predictive Tool for Primary Ciliary Dyskinesia. *Eur. Respir. J.* **2016**, *47*, 1103–1112, doi:10.1183/13993003.01551-2015.
3. Baz-Redón, N.; Rovira-Amigo, S.; Fernández-Cancio, M.; Castillo-Corullón, S.; Cols, M.; Caballero-Rabasco, M.A.; Asensio, Ó.; de Vicente, C.M.; del Mar Martínez-Colls, M.M.; Torrent-Vernetta, A.; et al. Immunofluorescence Analysis as a Diagnostic Tool in a Spanish Cohort of Patients with Suspected Primary Ciliary Dyskinesia. *J. Clin. Med.* **2020**, *9*, 3603, doi:10.3390/JCM9113603.
4. Baz-Redón, N.; Rovira-Amigo, S.; Paramonov, I.; Castillo-Corullón, S.; Cols Roig, M.; Antolín, M.; García Arumí, E.; Torrent-Vernetta, A.; de Mir Messa, I.; Gartner, S.; et al. Implementation of a Gene Panel for Genetic Diagnosis of Primary Ciliary Dyskinesia. *Arch. Bronconeumol.* **2021**, *57*, 186–194, doi:10.1016/J.ARBRES.2020.02.010.
5. Beydon, N.; Kouis, P.; Marthin, J.K.; Latzin, P.; Colas, M.; Davis, S.D.; Haarman, E.; Harris, A.L.; Hogg, C.; Kilbride, E.; et al. Nasal Nitric Oxide Measurement in Children for the Diagnosis of Primary Ciliary Dyskinesia: European Respiratory Society Technical Standard. *Eur. Respir. J.* **2023**, *61*, doi:10.1183/13993003.02031-2022.
6. Kempeneers, C.; Seaton, C.; Garcia Espinosa, B.; Chilvers, M.A. Ciliary Functional Analysis: Beating a Path towards Standardization. *Pediatr. Pulmonol.* **2019**, *54*, 1627–1638, doi:10.1002/PPUL.24439.
7. Landrum, M.J.; Lee, J.M.; Benson, M.; Brown, G.R.; Chao, C.; Chitipiralla, S.; Gu, B.; Hart, J.; Hoffman, D.; Jang, W.; et al. ClinVar: Improving Access to Variant Interpretations and Supporting Evidence. *Nucleic Acids Res.* **2018**, *46*, D1062–D1067, doi:10.1093/nar/gkx1153.
8. Kopanos, C.; Tsiolkas, V.; Kouris, A.; Chapple, C.E.; Albarca Aguilera, M.; Meyer, R.; Massouras, A. VarSome: The Human Genomic Variant Search Engine. *Bioinformatics* **2019**, *35*, 1978–1980, doi:10.1093/BIOINFORMATICS/BTY897.
9. Plagnol, V.; Curtis, J.; Epstein, M.; Mok, K.Y.; Stebbings, E.; Grigoriadou, S.; Wood, N.W.; Hambleton, S.; Burns, S.O.; Thrasher, A.J.; et al. A Robust Model for Read Count Data in Exome Sequencing Experiments and Implications for Copy Number Variant Calling. *Bioinformatics* **2012**, *28*, 2747–2754, doi:10.1093/bioinformatics/bts526.
10. Cameron, D.; Schröder, J.; Penington, J.; Do, H.; Molania, R.; Dobrovic, A.; Speed, T.; Papenfuss, A. GRIDSS: Sensitive and Specific Genomic Rearrangement Detection Using Positional de Bruijn Graph Assembly. *Genome Res.* **2017**, *27*, 2050–2060, doi:10.1101/gr.222109.117.
11. den Dunnen, J.T.; Dalgleish, R.; Maglott, D.R.; Hart, R.K.; Greenblatt, M.S.; Mcgowan-Jordan, J.; Roux, A.F.; Smith, T.; Antonarakis, S.E.; Taschner, P.E.M. HGVS Recommendations for the Description of Sequence Variants: 2016 Update. *Hum. Mutat.* **2016**, *37*, 564–569, doi:10.1002/humu.22981.
12. Richards, S.; Aziz, N.; Bale, S.; Bick, D.; Das, S.; Gastier-Foster, J.; Grody, W.W.; Hegde, M.; Lyon, E.; Spector, E.; et al. Standards and Guidelines for the Interpretation of Sequence Variants: A Joint Consensus Recommendation of the American College of Medical Genetics and Genomics and the Association for Molecular Pathology. *Genet. Med.* **2015**, *17*, 405–424, doi:10.1038/gim.2015.30.
13. Zhang, Q.; Giacalone, J.C.; Searby, C.; Stone, E.M.; Tucker, B.A.; Sheffield, V.C. Disruption of RPGR Protein Interaction Network Is the Common Feature of RPGR Missense Variations That Cause XLRP. *Proc. Natl. Acad. Sci. U. S. A.* **2019**, *116*, 1353–1360, doi:10.1073/pnas.1817639116.
14. Cols, N.; Marfany, G.; Atrian, S.; Gonzàlez-Duarte, R. Effect of Site-Directed Mutagenesis on Conserved Positions of Drosophila Alcohol Dehydrogenase. *FEBS Lett.* **1993**, *319*, 90–94, doi:10.1016/0014-5793(93)80043-T.

SUPPLEMENTARY FIGURES, VIDEOS AND TABLES

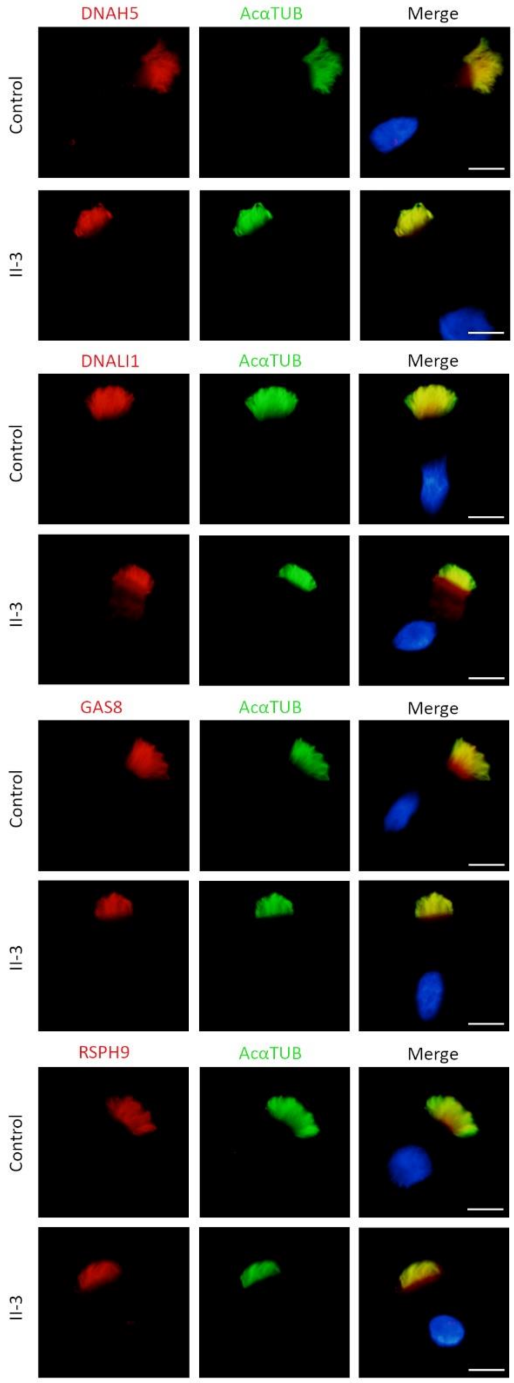

Figure S1: Immunofluorescence analysis of ciliary axoneme proteins in control and proband (II-3) nasal ciliated epithelia samples. Subcellular localisation of DNAH5, DNALI1, GAS8 and RSPH9 proteins (in red) in ciliary axoneme, observed by acetylated α-tubulin (AcαTUB, in green). The third column shows the final merged image with the nuclei stained with DAPI (in blue). Scale bar, 5 µm.

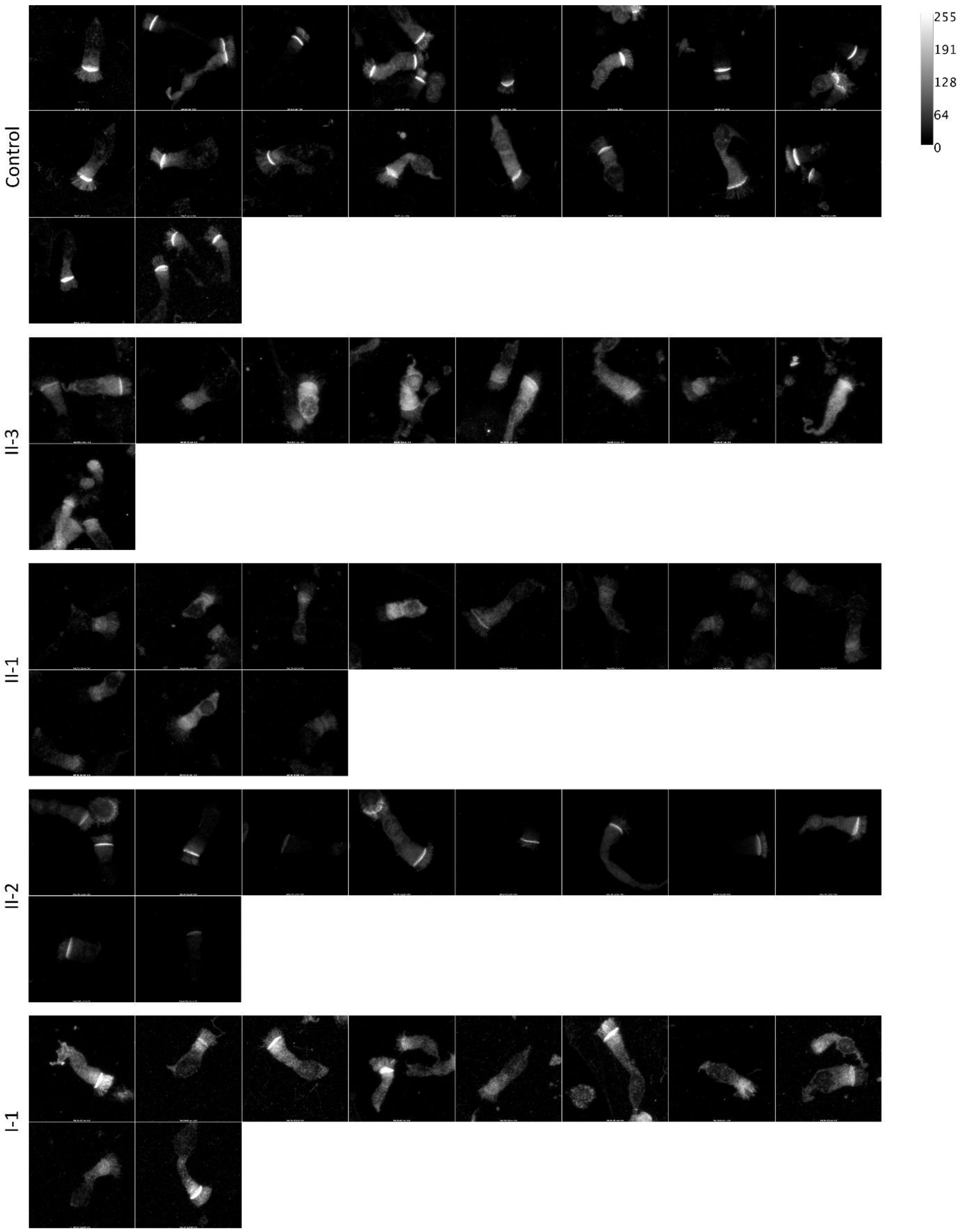

Figure S2: Analysed z-projections of RPGR protein immunofluorescence in nasal brushing samples. Ciliated cells from all family members showing localisation of RPGR protein in the transition zone. Z-projection Sum Slices of the anti-RPGR fluorescence channel was performed to measure the integrated density parameter (scale in upright corner).

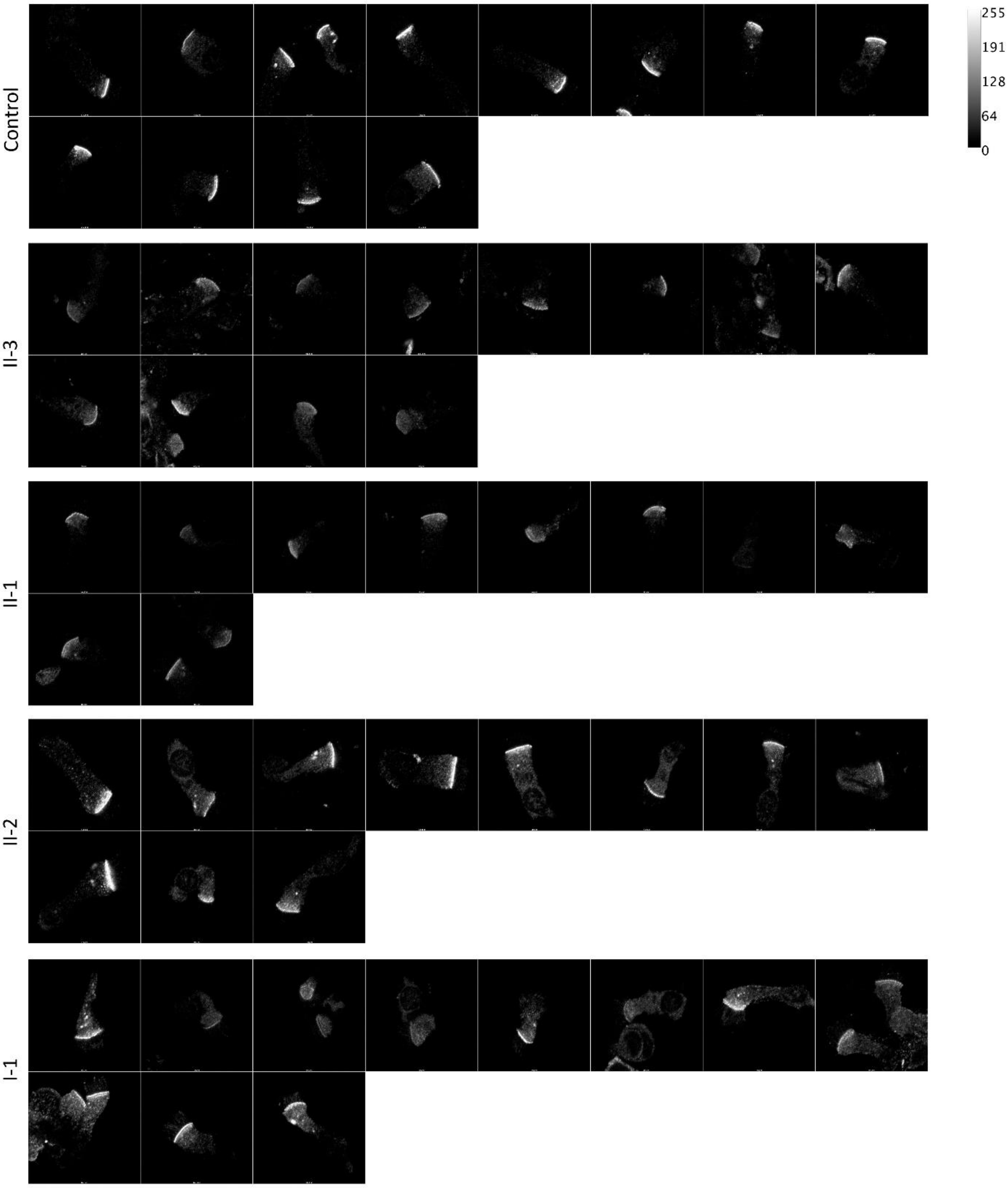

Figure S3: Analysed z-projections of CEP290 protein immunofluorescence in nasal brushing samples. Ciliated cells from all family members showing localisation of CEP290 protein in the transition zone. Z-projection Sum Slices of the anti-CEP290 fluorescence channel was performed to measure the integrated density parameter (scale in upright corner).

SUPPLEMENTARY VIDEOS:

Video S1: High-speed video-microscopy analysis (HSVM) in the nasal brushing sample of the proband (II-3). Example of video recording of proband (II-3) human nasal cells demonstrating the stiff and disorganised ciliary beat pattern. The video was recorded at 500fps using a 100x objective and was edited to a final 16x speed.

Video S2: High-speed video-microscopy analysis (HSVM) in the nasal brushing sample of the mother (I-1). Example of video recording of mother (I-1) human nasal cells demonstrating the stiff and disorganised ciliary beat pattern. The video was recorded at 500fps using a 100x objective and was edited to a final 16x speed.

Video S3: High-speed video-microscopy analysis (HSVM) in the nasal brushing sample of the brother (II-1). Example of video recording of brother (II-1) human nasal cells demonstrating the unaffected ciliary beat frequency and pattern. The video was recorded at 500fps using a 100x objective and video was edited to a final 16x speed.

Table S1: Candidate variants identified by whole exome sequencing (WES) in this family case (II-3 proband, II-1 brother, II-2 sister, I-1 mother, and I-2 father)

Genes	Variants (chromosome position)	Variants (ACMG nomenclature)	II-3	II-1	II-2	I-1	I-2	CADD[1]	ACMG classification[2]	OMIM entry[3]	ClinVar Id[4]
ADGB	6-147105887-T-C	NM_024694.4:c.4169T>C;p.Met1390Thr	0/1	0/0	0/1	0/0	0/1	25.6	VUS	614630	NA
ALG2	9-101983992-T-C	NM_033087.4:c.185A>G;p.Glu62Gly	0/1	0/0	0/0	0/1	0/0	27.6	VUS	601057	1500071
C4orf29	4-128942393-AGTA-	NM_001039717.2:c.699+1073_699+1076del	0/1	0/0	0/0	0/1	0/0	NA	VUS	-	NA
CDHR1	10-85968080-C-	NM_033100.4:c.1117del	0/1	0/0	0/1	0/1	0/0	NA	LP	609502	NA
CEP290	12-88530530-G-A	NM_025114.4:c.331C>T;p.Arg111Trp	0/1	0/0	0/1	0/0	0/1	30.0	VUS	610142	965275
DIS3	13-73333935-A-G	NM_014953.5:c.2875T>C;p.Ter959Glnext*14	0/1	0/0	0/0	0/0	0/1	14.1	LP	607533	1336735
ECHDC2	1-53370340-A-G	NM_001198961.2:c.680T>C;p.Leu227Pro	0/1	0/0	0/1	0/1	0/0	29.2	VUS	-	2343423
EIF4E3	3-71774435--G	NM_001134651.2:c.84dup;p.Glu29ArgfsTer15	0/1	0/0	0/0	0/0	0/0	NA	LP	609896	NA
EXOSC5	19-41897793-G-A	NM_020158.4:c.337C>T;p.Arg113Cys	0/1	0/0	0/0	0/1	0/0	34.0	VUS	606492	NA
FGGY	1-59978072-G-T	NM_018291.5:c.760G>T;p.Val254Phe	0/1	0/0	0/1	0/1	0/0	28.5	VUS	611370	NA
GDF5	20-34025074-T-A	NM_000557.5:c.631+4A>T	0/1	0/0	0/0	0/0	0/0	11.7	VUS	601146	NA
IRF2	4-185340725--TAA	NM_002199.4:c.88-3_88-2insTTA	0/1	0/0	0/0	0/0	0/0	NA	VUS	147576	NA
KRR1	12-75900397-AAA-	NM_007043.7:c.394-10_394-8del	0/2	0/0	2/3	1/2	0/0	NA	VUS	612817	NA
LZTR1	22-21351011-A-G	NM_006767.4:c.2246A>G;p.Tyr749Cys	0/1	0/0	0/0	0/0	0/1	28.1	VUS	600574	549754
MRPL37	1-54681899-T-C	NM_016491.4:c.1076T>C;p.Leu359Pro	0/1	0/0	0/1	0/1	0/0	27.6	VUS	611843	NA
NPEPPS	17-45669429--AAA	NM_006310.4:c.1365+9_1365+11dup	0/2	1/2	1/2	1/2	0/1	NA	VUS	606793	NA
OPLAH	8-145109753-C-T	NM_017570.5:c.2473G>A;p.Gly825Arg	1/1	0/1	0/0	0/1	0/1	34.0	VUS	614243	578462
PDS5A	4-39928487--A	NM_001100399.2:c.343-6dup	0/1	0/0	0/0	0/2	0/1	NA	VUS	613200	NA
PLXNB2	22-50719174-T-C	NM_012401.4:c.3992A>G;p.Asn1331Ser	0/1	0/0	0/1	0/0	0/1	13.7	LP	604293	NA
RNF126	19-648891-C-T	NM_194460.3:c.661G>A;p.Glu221Lys	0/1	0/0	0/0	0/1	0/0	33.0	VUS	615177	NA
RYR3	15-34130024-T-C	NM_001036.6:c.11843T>C;p.Met3948Thr	0/1	0/0	0/1	0/1	0/0	24.5	VUS	180903	NA
SCAPER	15-76958026--T	NM_020843.4:c.2613dup;p.Ala872SerfsTer13	0/1	0/0	0/0	0/0	0/0	NA	LP	611611	NA
SIGIRR	11-406453-G-A	ENST00000431843.2:c.965C>T;p.Thr322Met	0/1	0/0	0/0	0/1	0/0	27.3	VUS	605478	NA
SLC24A3	20-19193520-CGC-	NM_020689.4:c.54_56del;p.Arg20del	0/1	0/0	0/0	0/0	0/0	NA	VUS	609839	NA
SLC38A7	16-58704973-C-T	NM_018231.3:c.1207G>A;p.Ala403Thr	0/1	0/0	0/1	0/0	0/1	35.0	VUS	614236	2393905
SNED1	2-242021733-G-A	NM_001080437.3:c.4075G>A;p.Glu1359Lys	0/1	0/0	0/0	0/0	0/1	33.0	VUS	616634	NA

Table S1 (continued)

SOX9	17-70119713--CCCCCCCCCCAA	NM_000346.4:c.714_715ins;p.Pro238_Thr239ins	0/1	0/0	0/0	0/0	0/0	NA	VUS	608160	NA
SPRED3	19-38882864-CCT-	NM_001042522.2:c.381_383del;p.Ser128del	0/1	0/0	0/0	0/0	0/0	NA	VUS	609293	NA
STK36	2-219544451-A-G	NM_015690.5:c.947A>G;p.Lys316Arg	0/1	0/0	0/1	0/1	0/0	11.6	VUS	607652	NA
TECTA	11-120998763-G-A	NM_005422.4:c.2077G>A;p.Gly693Arg	0/1	0/0	0/0	0/0	0/1	24.9	VUS	602574	229293
TEP1	14-20848453-C-T	NM_007110.5:c.4944G>A;p.Trp1648Ter	0/1	0/0	0/1	0/1	0/0	36.0	VUS	601686	NA
TMEM82	1-16070695-T-C	NM_001013641.3:c.377T>C;p.Leu126Pro	0/1	0/0	0/1	0/1	0/0	25.8	VUS	-	NA
TNC	9-117788941-C-G	NM_002160.4:c.6203G>C;p.Gly2068Ala	0/1	0/0	0/0	0/1	0/0	24.7	VUS	187380	NA
TSHR	14-81528523-C-T	NM_000369.5:c.202C>T;p.Pro68Ser	0/1	0/0	0/1	0/0	0/1	24.0	VUS	603372	437071
UPK1A	19-36166829-T-C	ENST00000222275.2:c.556T>C;p.Trp186Arg	0/1	0/0	0/0	0/1	0/0	26.8	VUS	611557	NA
ZC3HC1	7-129663374-G-A	NM_016478.5:c.1210C>T;p.Arg404Cys	0/1	0/0	0/1	0/0	0/1	27.5	VUS	619746	NA

[1]CADD: Combined Annotation Dependent Depletion, candidate variants with a ≥15 score were considered. [2]Classification of variants according to ACMG guidelines (Varsome, https://varsome.com/); [3]OMIM entry (https://www.omim.org/). OMIM was used to look up described human genes and genetic phenotypes; [4]ClinVar identifier (https://www.ncbi.nlm.nih.gov/clinvar/); ACMG: The American College of Medical Genetics and Genomics; 0: wild-type allele; 1: mutated allele with the cited genetic variant; e; 2 or 3: mutated allele with another genetic change in the same position; VUS: variant of uncertain significance; LP: likely pathogenic variant; NA: non-available data.
